# *Alphafuser*: a parsimonious approach to predicting higher-order protein complexes

**DOI:** 10.1107/S2059798326003013

**Published:** 2026-04-23

**Authors:** Audrey Guillotin, Stephanie Hutin, Lorelei Masselot-Joubert, Kamel Hammani, Chloe Zubieta, Max Nanao

**Affiliations:** a Laboratoire de Physiologie Cellulaire et Végétale, Université Grenoble-Alpes, CNRS, CEA, INRAE, IRIG–DBSCI, 17 Rue des Martyrs, 38000Grenoble, France; b Institut de Biologie Moléculaire des Plantes, Centre National de la Recherche Scientifique (CNRS) Université de Strasbourg 12 Rue du Général Zimmer 67084Strasbourg France; c Structural Biology Group European Synchrotron Radiation Facility 71 Avenue des Martyrs 38000Grenoble France; Osaka University, Japan

**Keywords:** *AlphaFold*, complexes, multimers, interactome

## Abstract

Many biological processes rely on the activity of multiple proteins acting in concert as part of higher-order oligomeric complexes. However, the majority of structural studies have routinely simplified these systems to examine only one or a few protein partners of a multi-component system. Artificial intelligence (AI) methods implemented in *AlphaFold*, *RoseTTAFold*, *ESMFold* and others have revolutionized our ability to not only predict the fold of individual proteins, but also to predict that of complex assemblies. A major bottleneck in exploiting AI-based protein fold prediction for multiprotein complexes, however, is identifying likely interacting partners from a given interactome. Here, we present a protein complex prediction pipeline based on *AlphaFold*, called *Alphafuser*, that combines experimental interaction data with systematic querying of possible combinations of protein partners in a computationally parsimonious manner. This versatile protein complex prediction pipeline creates a queue of all potential complexes, up to a user defined number of partners. Because a complete search of all permutations is not typically practical, we implemented a simple dead-end trimming algorithm from the dimer step onwards based on the interface probability template modeling (ipTM) score to remove low-probability subcomplexes from the queue of higher-order complexes. Experimentally structurally characterized multiprotein complexes in the Protein Data Bank, PP2A-B56γ1 holoenzyme–PME-1 complex, the human γ-secretase complex, the TFIIIC complex and the TRAPP I complex, were used to obtain a general ipTM cutoff parameter for the pipeline and to confirm that complexes without extensive direct contacts between all subunits could be identified. We applied *Alphafuser* to two test cases starting from yeast two-hybrid and co-immunoprecipitation/mass spectrometry (co-IP/MS) interaction data. These predictions were tested experimentally using pull-down assays, confirming direct inter­actions of the proteins identified computationally by the *Alphafuser* pipeline.

## Abstract 2

*Alphafuser* is a structure-prediction pipeline that integrates experimental interaction data with *AlphaFold*-based modeling to systematically assemble multiprotein complexes in a computationally efficient manner. By implementing an ipTM-based pruning algorithm and validating against known structures and experimental assays, *Alphafuser* enables the accurate identification of higher-order assemblies from large interactome datasets.

## 1. Introduction

Stable and transient macromolecular complexes play a central role in fundamental biological processes from signal transduction to cell fate (Westermarck *et al.*, 2013 ▸; Staley & Woolford, 2009 ▸; Pawson & Nash, 2000 ▸). An understanding of the molecular basis underlying cellular function and disease pathology often requires high-resolution structure determination. However, very little of the protein–protein interactome (PPI) has been structurally characterized, with estimates of less than 4% of the human PPI determined for binary complexes (Luck *et al.*, 2020 ▸; Lee *et al.*, 2024 ▸). Experimental, *in silico* and combinations of both approaches have sought to identify the protein partners in multiprotein complexes and to provide the biological context of these macromolecular assemblies (Uetz *et al.*, 2000 ▸; Spirin & Mirny, 2003 ▸; Marsh *et al.*, 2013 ▸; Totir *et al.*, 2012 ▸). However, even with improved experimental setups including extremely high flux synchrotron beamlines, free-electron lasers, more powerful electron microscopes and highly sensitive mass spectrometers, sample preparation, data collection, analysis and structure building remain significant bottlenecks. Combining new protein fold prediction methods (Jumper *et al.*, 2021 ▸; Abramson *et al.*, 2024 ▸; Baek *et al.*, 2021 ▸; Lin *et al.*, 2023 ▸; Evans *et al.*, 2022 ▸; Zheng *et al.*, 2021 ▸; Lee *et al.*, 2023 ▸) with experimentally determined and validated protein–protein inter­actions facilitates the prediction of physiologically relevant multiprotein complexes that have not been directly observed.

*AlphaFold* [*AlphaFold*2 (Jumper *et al.*, 2021 ▸), *AlphaFold Multimer* (Evans *et al.*, 2022 ▸), *AlphaFold*3 (Abramson *et al.*, 2024 ▸), collectively called AF] has been a major advance in addressing the protein-folding and protein-interaction prediction challenges in structural and computational biology. The demonstrated high accuracy of these methods for predictions of folding for single polypeptide chains and small multimeric complexes suggest that predicting the components and overall conformation of biologically relevant multiprotein complexes is feasible. Recent applications of AF in virtual yeast two-hybrid assays and two-partner interaction screening have demonstrated a high degree of overlap between prediction and experiment (Lee *et al.*, 2024 ▸; Bryant *et al.*, 2022 ▸). As predictions from AF are able to be ranked by probability criteria, the most likely, and potentially physiologically relevant, complexes may be identified. While AF-based fold prediction may not be sufficient to identify atomic-resolution interactions (Terwilliger *et al.*, 2024 ▸), it is likely to be accurate enough for the identification of multi-component complexes of interest, which can then be targeted for experimental structure determination.

A major limitation in the large-scale prediction of interactomes is the computationally intractable number of different polypeptide combinations for even a small interactome. For example, with *n* identified partners, assuming *r* partners in a complex, the number of different potential complexes is *nCr* = *n*!/*r*!(*n* − *r*)!. For a ten-protein interactome with dimer to decamer complexes this corresponds to ∼1400 different possibilities, and for a 40-member interactome over a trillion possibilities. Because the actual interactome, even including transient complexes, is likely to populate only a small part of this combinatorial space, we reasoned that we could use the interface probability template modeling (ipTM) score to iteratively sparsify the search space and identify biologically interesting complexes in a computationally tractable fashion.

**Figure 1:**
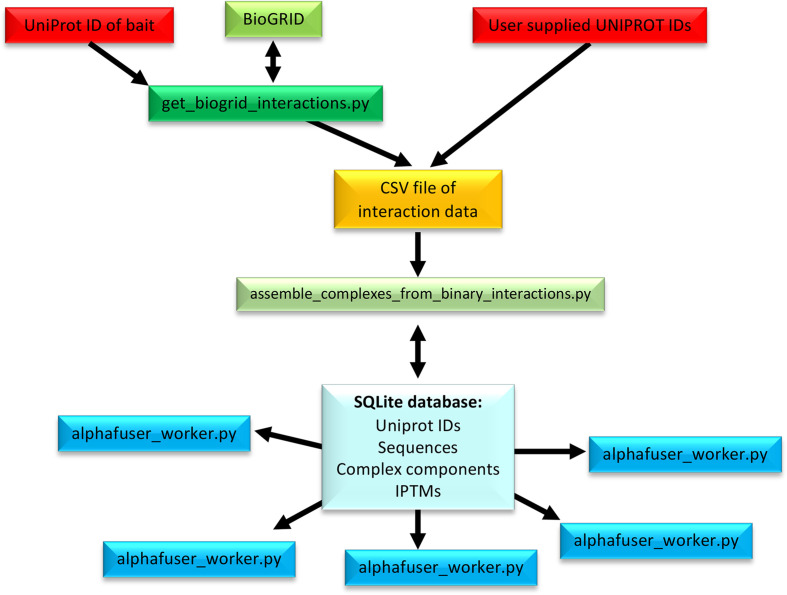
Schematic overview of the *Alphafuser* workflow. The UniProt ID of the target protein of interest is used to generate an interactome for *Alphafuser*. This step can be substituted for a user-defined interactome file in CSV format. A binary interactome is then assembled and fed into *Alphafuser* using the alphafuser_worker.py script. Monomeric structures are downloaded from EMBL–EBI or generated.

Here, we present *Alphafuser*, a structure-prediction pipeline workflow (Fig. 1 ▸), training case examples and two test cases using experimental data of (i) a large yeast two-hybrid data set of 250 transcription-factor (TF) interactions in *Arabidopsis* gynoecium development (Herrera-Ubaldo *et al.*, 2023 ▸) and (ii) a 40-member interactome from co-immunoprecipitation/mass spectrometry (co-IP/MS) experiments on a chloroplast RNA-binding protein (Méteignier *et al.*, 2021 ▸). Yeast two-hybrid experiments, one of the more common methods for querying protein–protein interactions, performed as a matrix may yield hundreds of binary interactions, but provide no information as to higher-order complex formation. Applying AF predictions based on a set of experimentally determined dimeric complexes allows the probability-based generation of larger complexes that may be functionally important in a physiological context and in different biological processes. In the case of co-IP/MS, these data provide direct and indirect interaction partners of the protein of interest but often contain artefactual proteins that are not true interactors or only interact indirectly. By ranking the probabilities of possible subcomplexes, putative physiologically relevant protein assemblies, containing a protein of interest, may be identified based on AF predictions. For a given interactome, all possible dimers are first generated in *Alphafuser*, ranked based on ipTM score. If the ipTM score is above a user-defined threshold, these scores are propagated in the form of priorities to supercomplexes containing these dimers. Supercomplexes containing only low-scoring composite dimers are removed from the queue. Supercomplexes containing the highest scoring dimers are calculated first. In this way, the number of higher-order complexes to compute is drastically reduced and the most probable complexes are prioritized. Using the *Alphafuser* output, we experimentally validated a number of predicted interactions via pull-down assays, suggesting that the AF-based *Alphafuser* pipeline provides a convenient tool for the identification of multimeric protein complexes based on experimental interactome data.

## 2. Methods

### 2.1. Software workflow

*Alphafuser* is a collection of Python and Perl scripts, with the code available on GitHub (https://github.com/mnanao/alphafuser). A workflow overview is shown in Fig. 1 ▸. Interaction data may be user-supplied experimental data or derived from databases such as BioGRID (Oughtred *et al.*, 2021 ▸) and input as a text file in CSV format using the protein UniProt ID (UniProt Consortium, 2023 ▸). It should be noted that although numerous databases of protein sequences exist, *Alphafuser* uses UniProt identification numbers to allow facile mapping to monomeric AF2 structures using a unique identifier. Once a CSV file of interaction data is obtained, the list of supercomplexes is assembled and used to populate an SQLITE database. Protein sequences associated with UniProt IDs are inserted into this database. Optionally, proteins are trimmed to high-confidence regions using *phenix.process_predicted_model* (Oeffner *et al.*, 2022 ▸), which removes poorly structured regions based on a user-defined pLDDT score cutoff. Finally, complex structures are computed using an *Alphafuser* worker script, which itself creates input data for *LocalColabFold* (Mirdita *et al.*, 2022 ▸). The worker script may be executed in parallel, although only primitive handling of database access concurrency is implemented, and has not been used with more than ten concurrent worker instances. Wall time limits are set on a per GPU-type basis, and considerable attrition may occur on higher-order complexes due to longer run time, insufficient memory or target-specific problems. *Alphafuse*r simulations were performed at the ESRF SLURM (Yoo *et al.*, 2003 ▸) computer cluster, requesting GPUs with at least 48 Gb of RAM, which at the time of computations consisted of 50 A40, 14 A100 and two H100 NVIDIA GPUs.

### 2.2. BioGRID interactome data

In order to facilitate the automatic identification of potential binding partners of a protein of interest, we have written a Python script to query the BioGRID database (Oughtred *et al.*, 2021 ▸). Only physical interaction criteria are accepted by the script. Of the set of physical interactions, it is possible to filter by the number of reported physical interactions as well as by specific types of interaction data, such as co-purification and affinity capture. Bearing in mind that the scale and scope of interaction data in this database vary widely per organism, it is not possible to provide general criteria applicable to all systems. However, because of the existence of several organism-wide affinity-capture studies, we have found that these interaction types are a good filter to start with, especially in well studied organisms such as humans, yeast and mice. The selection of criteria for interactions to be accepted must also take into account that the number of combinations increases with the power of the number of proteins.

### 2.3. Sequence trimming

Once the queue has been assembled in the SQLITE file, an optional filtering step can be performed on the sequences. Specifically, the UniProt IDs are used to query the EMBL–EBI AF database (Varadi *et al.*, 2024 ▸). It is important to note that while there is enormous coverage of sequences in this database, some proteins (for example extremely large proteins) do not have models. If a model is not present in the EMBL–EBI AF database, it is generated within the *Alphafuser* pipeline. If present, the AF prediction for a monomer is downloaded and is optionally processed by *Phenix* (Zwart *et al.*, 2008 ▸) to excise low-confidence regions. This approach was implemented to reduce computational time. However, some interactions could be lost in cases where low-confidence regions transition to high-confidence regions in the context of partner-protein interactions, for example.

### 2.4. Supercomplex filtering

The number of complexes to test becomes computationally untenable with even a moderate number of binary interactions as a starting input. We have therefore developed a simple system for the dynamic pruning of complexes, once an initial list has been established. We reasoned that pairwise inter­actions that are not confirmed by AF2 could be propagated to larger complexes containing that interaction. That is, if *A*–*B* scores poorly, *A*–*B*–*C* and *A*–*B*–*C*–*D* could be removed from the list of complexes to evaluate. However, this assumes that supercomplex components all interact directly and equally well. While this was the initial concept for this work, and is quite common at least in lower-order complexes, this is not always the case. The simplest exception to this assumption would be a bridging arrangement of proteins in which a relatively small binary interface is stabilized by a third partner. In this case, supercomplexes of interest could be erroneously eliminated. In order to mitigate this issue, two methods are employed. Firstly, no removal of complexes is performed until all pairwise evaluations are complete, and secondly, a prioritization step is used. Specifically, the workflow and prioritization include three steps.

(i) Dimer evaluation. For each dimer, if the ipTM score is above the priority threshold (a user-defined ipTM value; usually between 0.5 and 0.8), supercomplexes containing the dimer are given a priority score equal to the ipTM score. If a supercomplex already has an associated priority, the priority will be reset to the new priority if it is higher than the existing priority. For example, if a complex *B*–*C* has an ipTM score of 0.6 and *B*–*C* appears in the complex *A*–*B*–*C*, where the complex *A*–*B*–*C* has a priority of 0.55 (*i.e.A*–*B* has an ipTM score of 0.55 and was initially set for the larger complex *A*–*B*–*C*), the complex *A*–*B*–*C* will be given the higher threshold value of 0.6 due to the presence of the *B*–*C* dimer.
(ii) Once all dimers have been evaluated, supercomplex pruning is performed. Each supercomplex with less than the priority threshold score is marked for removal. This removes any complexes that contain only eliminated dimers from the queue. This step is referred to as dead-end trimming or dead-end elimination and is listed as ‘poisoned’ in the *Alphafuser* output.
(iii) For trimers and higher, complexes are evaluated in order of priority based on the ipTM score. This score is propagated upwards to any larger complex in the queue that contains the evaluated complex, enabling dynamic removal from the queue of complexes containing subcomplexes that score below a given threshold value and with computation of complexes with higher scores performed first. All *n*-mers are calculated before moving to higher-order complexes.

### 2.5. Experimental validation

29 targets were selected for cloning, protein production and interaction assays. For the yeast two-hybrid data, low-scoring binary interactions were tested to determine whether false positives were prevalent in the data. All binary interactions, even with ipTM scores of 0.2, yielded positive interactions by pull-down. For mTERF9, the highest scoring binary inter­actions were tested. For both datasets, the top 10% of trimeric complexes were examined and targets were preferentially selected if they appeared multiple times in the list in order to minimize cloning and protein-production steps while allowing us to examine multiple different combinations. Attrition due to failed cloning, low protein production from *in vitro* transcriptional translation and nonspecific binding of the HA-tagged proteins to the anti-FLAG magnetic beads eliminated some targets from testing (Supplementary Table S1). Target genes were cloned into appropriate expression vectors with N-terminal or C-terminal tags for downstream analysis.

### 2.6. Target cloning

*AS2* (AT1G65620.1; UniProt O04479), *BEE1* (AT1G18400; UniProt Q8GZ13), *BP* (AT4G08150; UniProt P46639), *CUC1* (AT3G15170; UniProt Q9FRV4), *FIL* (AT2G45190; UniProt O22152), *HEC1* (AT5G67060.1; UniProt Q9FHA7), *HEC2* (AT3G50330; UniProt Q9SND4), *IND* (AT4G00120.1; UniProt O81313), *KAN2* (AT1G32240.1; UniProt Q9C616), *KNAT6* (AT1G23380; UniProt Q84JS6), *LUG* (AT4G32551; UniProt Q9FUY2), *NGA3* (AT1G01030.1; UniProt Q9MAN1), *PNF* (AT2G27990.1; UniProt Q9SJJ3), *RPL* (AT5G02030; UniProt Q9LZM8), *SPT* (AT4G36930.1; UniProt Q9FUA4), *STM* (AT1G62360.1; UniProt Q38874), *YAB3* (AT4G00180.1; UniProt Q9XFB1), *WUS* (AT2G17950.1; UniProt Q9SB92), *mTERF9* (AT5G55580.1; UniProt Q9FM80), *ATHCF1BETA* (ATCG00480.1; UniProt P19366), *ATRAB8D* (AT4G20360.1; UniProt P17745 and B9DHZ8), *RSP19* (ATCG00820.1; UniProt P56808), *BL17C* (AT3G54210; UniProt Q9M385), *CPFTSY* (AT2G45770; UniProt O80842), *EL30X* (AT3G18740; UniProt Q9LSA3), *P2Y* (AT2G27710; UniProt Q9SLF7), *PXRQ* (AT3G26060; UniProt Q9LU86), *RPL22* (ATCG00810; UniProt P56795) and *US10Y* (AT3G47370.1; UniProt Q9STY6 and A0A178VBU3) cDNA were PCR-amplified using specific primers and inserted into the *pTnT* (Promega L5610) vector. Primers used to generate the constructs are listed in Supplementary Table S1. For pull-down experiments, the following C- or N-terminally tagged constructs were used: pTnT-C-3×FLAG, pTnT-N-5×Myc, pTnT-N-StrepTagII and pTnT-N-V5. Constructs are summarized in Supplementary Table S1.

### 2.7. Protein production

The vectors listed above were used for *in vitro* protein production using the TnT SP6 High-Yield Wheat Germ Protein Expression System (Promega, catalogue No. L3260) according to the manufacturer’s instructions. Briefly, *Escherichia coli* DH5α cells were transformed with the plasmid of interest and a single colony was used to inoculate a 15 ml overnight culture. The cells were centrifuged and the plasmid DNA was isolated using a QiaPrep Spin Miniprep Kit (Qiagen, catalogue No. 27104) according to the manufacturer’s instructions. 1 µg purified plasmid was used as input in a 35 µl reaction (1 µg plasmid, 20 µl TnT SP6 High-Yield Wheat Germ Master Mix, completed to 35 µl with nuclease-free water) incubated at 25°C for 2 h. To test binary and ternary interactions, 1 µg of each purified plasmid was used. 5 µl of each reaction was used as the input for Western blots. The rest of the reaction was used in pull-down experiments.

### 2.8. Pull-down assays and Western blotting

The TnT reaction solution was completed with 100 µl pull-down buffer (10 m*M* Tris pH 7.5, 300 m*M* NaCl, 1% Triton X-100, 1.5 mg ml^−1^ BSA) and 10 µl anti-FLAG magnetic beads (Anti-FLAG M2 magnetic beads; catalogue No. M8823, Merck Millipore). Following 1 h of incubation at 4°C, the anti-FLAG magnetic beads were immobilized on a magnetic rack and washed for 5 × 2 min under agitation with 500 µl pull-down buffer. Finally, the beads were resuspended in 20 µl phosphate-buffered saline and 10 µl SDS–PAGE loading dye and boiled for 5 min at 95°C. Western blots were used to verify the presence of protein complexes using anti-FLAG [monoclonal ANTI-FLAG M2-peroxidase (HRP) antibody; catalogue No. A8592, Sigma-Aldrich; 1:10 000 dilution], anti-Myc (Myc Tag Monoclonal Antibody, HRP; catalogue No. R951-25, Invitrogen; 1:5000 dilution), anti-HA (Anti-HA-Peroxidase High Affinity; catalogue No. 12013819001, Roche; 1:5000 dilution), anti-StrepTagII (StrepTag II Antibody HRP Conjugate; catalogue No. 71591-M, Sigma-Aldrich; 1:5000 dilution) and anti-V5 (V5 Tag Monoclonal Antibody, HRP; catalogue No. R961-25, Invitrogen; 1:10 000 dilution) antibodies.

## 3. Results

### 3.1. Parameterization of *Alphafuser*

**Figure 2:**
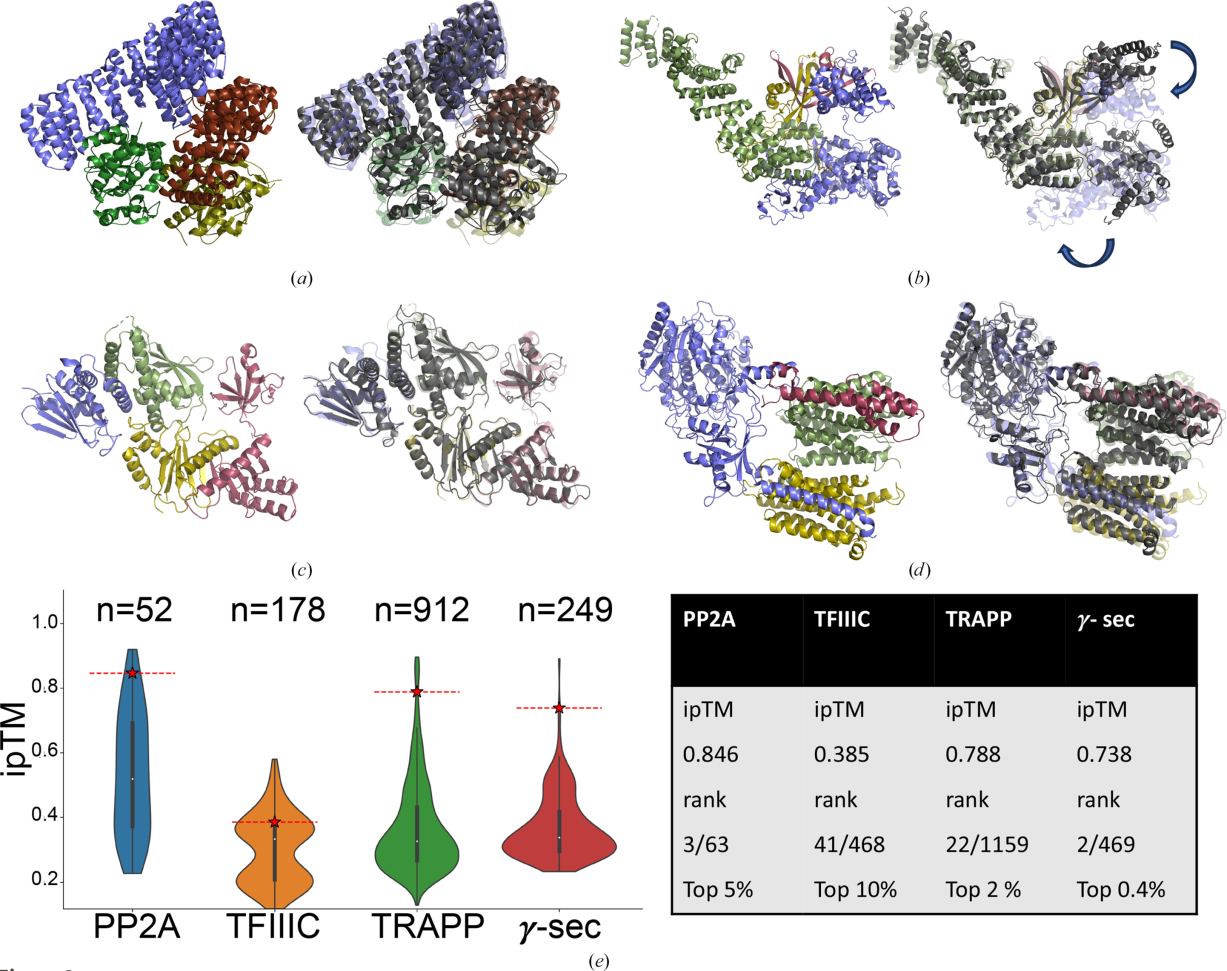
Experimental and predicted oligomeric structures used as a benchmarking set for *Alphafuser*. (*a*) Left, PP2A (PDB entry 7soy) with subunits colored uniquely. Right, overlay with the *Alphafuser*-predicted structure in dark gray. (*b*) TFIIIC TauA (PDB entry 8clk) colored as per (*a*). Right, arrows indicate the subunit rotations required to overlay the experimental and predicted structures. (*c*) TRAPP I (PDB entry 2j3t) colored as per (*a*). (*d*) γ-Secretase (PDB entry 6lr4) colored as per (*a*). Experimental structures are shown in partial transparency on the right for clarity. (*e*) Plot of ipTM scores for the test cases with the predicted structure corresponding to the experimental structure highlighted in red. In all cases, the experimental structure appeared within the top 10% of predicted complexes ranked by ipTM score.

In order to parameterize the *Alphafuser* pipeline, four multimeric complexes were used. These complexes, the PP2A-B56 γ1 holoenzyme–PME-1 complex (PDB entry 7soy; Li *et al.*, 2022 ▸), TFIIIC TauA (PDB entry 8clk; Seifert-Davila *et al.*, 2023 ▸), TRAPP I (PDB entry 2j3t; Kim *et al.*, 2006 ▸) and γ-secretase (PDB entry 6lr4; Yang *et al.*, 2021 ▸), have experimentally determined 3D structures and physical interaction data curated in BioGRID. Because only a few thousand complexes can be inferred on our cluster in a reasonable timeframe (on the order of weeks depending on the specific GPUs used and node availability), only affinity-capture protein–protein interaction data were selected and genetic interactions and PDB interactions were excluded (the latter because the test complexes were initially identified in the PDB). Due to differences in biochemical interactome coverage, the number of possible complexes varied for the test cases. The presence of all structurally determined partners was confirmed for each target case; however, in the case of the TFIIIC complex the positioning of one subunit was poorly predicted (Fig. 2 ▸). Table 1 ▸ lists the bait protein used for BioGRID, the selection criteria employed and the number of complexes in the expanded list of complexes that were calculated during the *Alphafuser* run.

### 3.2. PP2A–PME-1

Protein phosphatase 2A (PP2A) is the most abundant serine/threonine phosphatase in mammalian cells, playing roles in the cell cycle, apoptosis, metabolism and tumor suppression. It is composed of three subunits: a scaffolding subunit A (589 amino acids), a catalytic subunit C (309 amino acids) that forms the PP2Ac dimer, and a regulatory subunit B (449 amino acids). The activity of the complex is tightly regulated by reversible carboxymethylation of the PP2Ac tail modulated by protein phosphatase methylesterase, PME-1 (386 amino acids). The four-protein PP2A–PME-1 complex determined by electron microscopy (PDB entry 7soy) was used as a test case (Li *et al.*, 2022 ▸). This test case was also chosen because there are no direct contacts between subunit A and PME-1, which allowed us to test the efficacy of the priority thresholding and its ability to retain linear complexes.

The starting PP2A interactome was generated by selecting the protein phosphatase II regulatory subunit A2 and querying BioGRID for physical interaction partners (UniProt ID P67775 for subunit A2 was used as bait to limit the number of interactions). Ten interactors were identified and their sequences were downloaded and used as input for *Alphafuser*. The entire queue contained 63 complexes. ipTM thresholds of 0.5–0.9 were used and the target complex was calculated at all threshold levels. Although high-accuracy atomic models of complexes are not required for *Alphafuser* to succeed, it is worth noting that the AF model had an r.m.s.d. of 0.42 Å compared with the deposited structure (Fig. 2 ▸*a*). The predicted model additionally had a high ipTM score of 0.848. This suggests that rather high-priority thresholds could be applied, dramatically reducing the number of required computations. The *Alphafuser* structure with the lowest r.m.s.d. compared with the experimentally determined structure (PDB entry 7soy) was the third-ranked complex of 52 (with ten complexes deprioritized and one failed job).

Due to the relatively small size of the queue for this system resulting from the limited interactome, we used it to evaluate the effects of trimming sequences based on pLDDT scores by running the entire pipeline with both trimmed and untrimmed sequences. A pLDDT threshold of 0.7 was used to remove low-probability amino acids. This procedure removed 15.9% of amino acids from the dataset. A significant improvement in run time per complex was observed, from an average of 48 min for untrimmed down to 30 min for the trimmed sequences. Furthermore, comparing the top 20% of complexes by ipTM scores of the trimmed and untrimmed datasets, we found that 90% of the complexes are the same. Interestingly, for trimmed data, the predicted structure corresponding to the experimental structure was the third-ranked complex by ipTM, while for the untrimmed data it was the ninth-ranked structure, with an ipTM of 0.833 (Fig. 2 ▸ and Supplementary Data S1).

### 3.3. TFIIIC TauA

The human TFIIIC complex, comprising TauA and TauB subcomplexes, recruits RNA polymerase III to its tRNA target genes. The TauA subunits, TFIIIC35 (213 amino acids), TFIIIC63 (533 amino acids) and TFIIIC102 (886 amino acids), and the TauB subunit, TFIIIC220 (2158 amino acids), were used as a second test case (Seifert-Davila *et al.*, 2023 ▸). The initial interactome was assembled based on physical inter­action data for the 35 kDa α subunit with the starting interactome containing 14 different polypeptides (Table 1 ▸). Expansion of these interactions led to a queue of 468 complexes, which were then trimmed based on the previously discussed protocol in which unstructured low-probability regions were removed (29% of the residues were removed). The resulting *Alphafuser* model, consisting of three subunits of TauA and one subunit of TauB, had an ipTM of 0.385 and, although the global topology was consistent with the experimental structure (PDB entry 8clk), it differed significantly in some regions (predominantly the placement of the TFIIIC220 subunit) with an overall r.m.s.d. of 2.66 Å (Fig. 2 ▸*b*). Rerunning the *Alphafuser* pipeline with untrimmed data did not result in improved placement of this subunit. Because of the quite low ipTM score of the target complex (0.379) and dimeric complexes (average dimeric score of 0.399) only a priority threshold of 0.5 yielded runs with the target complex. Despite the overall quite low ipTM scores of the dataset, examination of the ipTM scores of the subdimers of this target complex that were present in the BioGRID pull-down data reveal high ipTM scores for TFIIIC220–TFIIIC63 (ipTM = 0.705), TFIIIC102–TFIIIC63 (ipTM = 0.763) and TFIIIC63–TFIIIC35 (ipTM = 0.52) (Supplementary Data S1).

### 3.4. TRAPP I

Transport protein particle (TRAPP I) is a vesicle-tethering factor that localizes to the Golgi and is involved in endoplasmic reticulum (ER) to Golgi transport via binding of ER-derived vesicles for subsequent fusion with the Golgi. The mammalian TRAPP I complex used as a test case consists of four subunits: BET3/TRAPP3C (182 amino acids), TRS33/TRAPPC6A (159 amino acids), BET5/TRAPPC1 (145 amino acids) and TRS23/TRAPPC4 (219 amino acids). As with PP2A, this complex was also selected because it lacks direct interactions between TRS33/TRAPPC6A and TRS23/TRAPPC4. The initial interactome was based on physical interaction data for the BET3/TRAPP3C subunit with the starting interactome containing 25 different polypeptides, which expanded to 1156 potential complexes (Table 1 ▸). The trimming procedure removed 24% of all residues. ipTM priority threshold values across the range resulted in the calculation of the target complex, with the exception of only the very highest value (0.9) (Table 2 ▸ and Supplementary Data S1). The predicted complex yielded a high ipTM (0.788) with an r.m.s.d. of 0.971 Å compared with the deposited structure (PDB entry 2j3t; Fig. 2 ▸).

### 3.5. γ-Secretase

Human γ-secretase is a multi-subunit membrane-embedded protease complex that cleaves various proteins, including amyloid precursor protein (APP). γ-Secretase is composed of four polypeptide subunits: the catalytic presenilin (PS1; 467 amino acids) subunit, presenilin enhancer 2 (Pen-2; 143 amino acids), anterior pharynx defective 1 (APH-1; 265 amino acids) and nicastrin (NCT; 709 amino acids) (Yang *et al.*, 2021 ▸). There are no direct connections between APH-1 and Pen-2. The initial interactome was based on physical interaction data for the APH-1 protein with the starting interactome containing 17 different polypeptides, expanded to 469 complexes (Table 1 ▸). Sequence trimming removed 22% of the residues. The *Alphafuser* model yielded a structure with an ipTM of 0.738 and an r.m.s.d. of 1.44 Å. ipTM priority thresholds of 0.5–0.8 all produced the target complex (Table 2 ▸ and Supplementary Data S1).

### 3.6. *Alphafuser* complex prediction and validation

**Figure 3:**
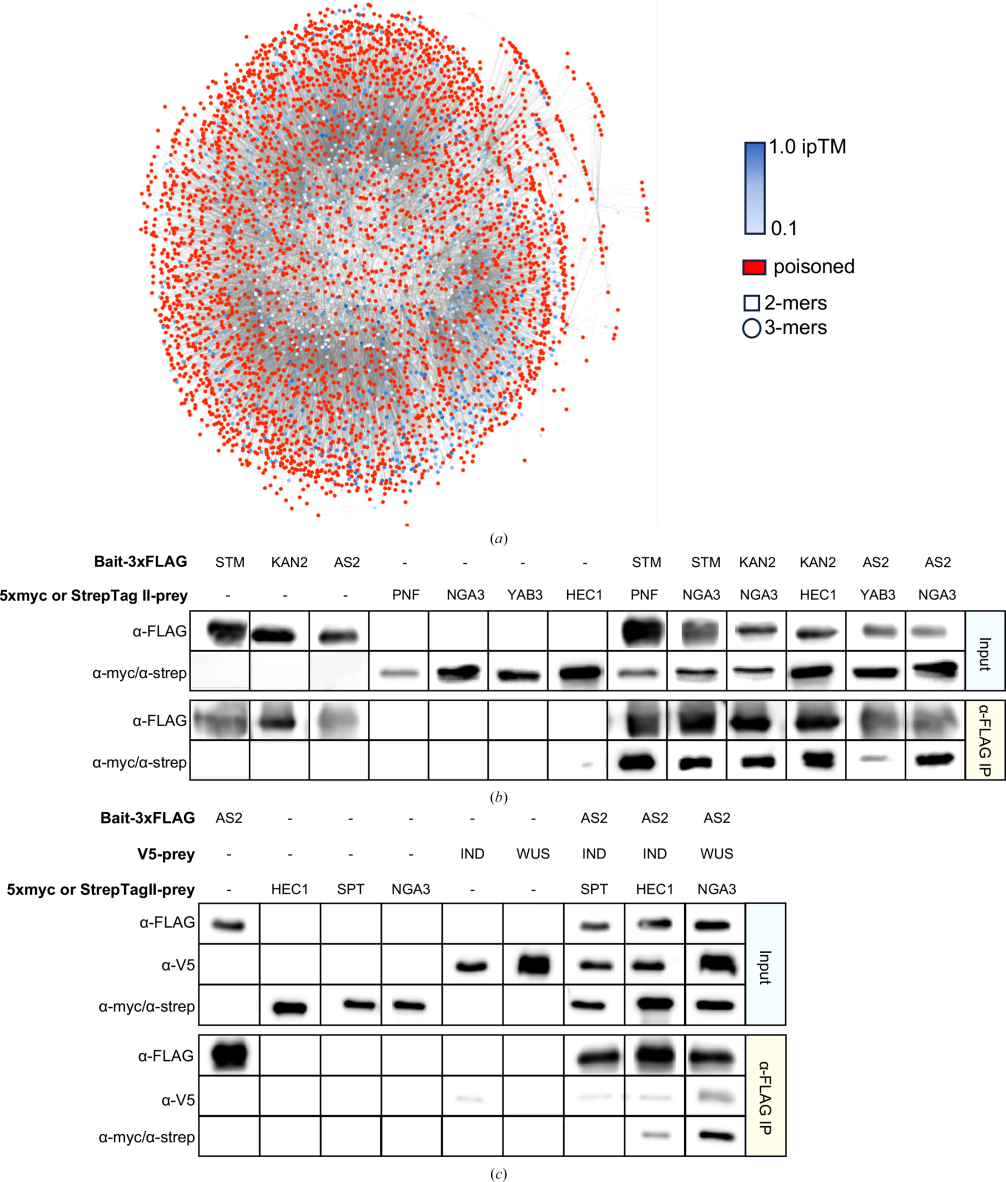
Network diagram and pull-down assays for the gynoecium interactome. (*a*) Dimer, trimer and tetramer complexes queued for computation from yeast two-hybrid data. Red denotes poisoned complexes based on an ipTM cutoff of 0.5. Dimers (squares), trimers (circles) and tetramers (triangles) are shown and are colored by ipTM, with darker blue denoting higher ipTM scores. (*b*) Pull-down experiment demonstrating interaction between gynoecium dimers. All proteins were expressed using *in vitro* transcription translation and different N- or C-terminal tags. (*c*) Pull-down experiment demonstrating interaction between gynoecium trimers. SPT was poorly expressed and its interactions with AS2 and IND could not be confirmed.

Based on the successful application of *Alphafuser* to known experimentally validated structures for the test cases set above, two protein interactome datasets from (i) yeast two-hybrid data and (ii) immunoprecipitation mass-spectrometry experiments, without experimental structural data, were used in the *Alphafuser* pipeline. A large yeast two-hybrid matrix screen of TF involved in *Arabidopsis thaliana* gynoecium development (Herrera-Ubaldo *et al.*, 2023 ▸) was used to predict the formation of higher-order complexes based on initial experimentally determined binary interactions. No target protein was used (default parameters) and the complex size was limited to trimers in order to limit the number of complexes to be computed. Starting from 368 binary inter­actions, leading to a queue of 4504 trimeric complexes, structure predictions were performed with *Alphafuser* using an ipTM priority cutoff of 0.5. Interestingly, many binary interactions confirmed as positive by yeast two-hybrid experiments exhibited average ipTM scores below 0.5, ranging from less than 0.2 to 0.7. In order to determine whether the data contained a large number of false positives, three very low scoring binary interactions were tested (ipTM < 0.25), two low-scoring ipTM interactions (ipTM 0.29–0.37) and one moderate-scoring interaction (ipTM 0.411). All binary complexes gave positive results in pull-down assays, consistent with the yeast two-hybrid data (Fig. 3 ▸). For this set of proteins, it should be noted that 59% of the residues, on average, were predicted to be disordered based on pLDDT scores (pLDDT < 0.7 or r.m.s.d. greater than 1.5; Oeffner *et al.*, 2022 ▸). For this reason, we ran *Alphafuser* without trimming sequences (Supplementary Data S1). It is not clear why the overall ipTM scores were lower for this set of proteins compared with the training sets; however, many plant TFs have extensive disordered regions and secondary-structural elements connected by relatively long random coils, which result in lower overall pTM scores for each component and lower ipTM scores for the complexes. Using a 0.5 ipTM cutoff, 3783 complexes were eliminated based on failing priority score cutoffs, and 719 complexes were calculated (two could not be calculated by AF). In visually inspecting the results of this run, we discovered that many complexes with relatively high ipTM scores yielded qualitatively unrealistic structures. Examination of the output thumbnail structures and predicted alignment error (PAE) matrices was consistent with this observation. Using the targets from the binary tests to minimize additional cloning steps and three additional targets, IND, WUS and SPT, three different high scoring trimers (ipTM > 0.4) were tested and one of the three (AS2/WUS/NGA3) gave clear positive results, one gave weakly positive bands (AS2/IND/HEC1), which we considered inconclusive, and one complex gave a clear negative result (AS2/IND/SPT). Of note, the complexes AS2/WUS/NGA3 (ipTM 0.422) and AS2/IND/HEC1 (ipTM 0.417) had lower scores than AS2/IND/SPT (ipTM 0.508). However, the pLDDT and pTM scores were higher for the detected complexes, while the PAE matrices were of similar quality for all three tested trimers. All trimers tested were in the top 10% of ipTM scores.

A second experimental dataset based on co-IP mass-spectrometry data was used to predict direct interactors of a target protein, mTERF9 (command-line option trim_all_complexes_to_include gene name). This protein is a member of the mitochondrial transcription termination factor (mTERF) family and is localized to the chloroplast. mTERF9 and its interactome are well structured compared with the previous example, with only 22% of residues falling below a pLDDT of 0.7, and this may correlate with the core metabolic role of the protein (Xie *et al.*, 2007 ▸). Proteins with different cellular functions will exhibit varying degrees of disorder, with eukaryotic TFs exhibiting a high prevalence of disordered regions versus other cellular proteins (Liu *et al.*, 2006 ▸). In contrast, proteins encoded by prokaryotic or prokaryotic-derived genes, as is the case for the plastid genomes of mitochondria and the chloroplast, exhibit fewer disordered regions and include a number of interacting partners of mTERF9 (Bhattacharya & Medlin, 1995 ▸; Falcón *et al.*, 2010 ▸). Due to the well folded interactome, trimming was used in the *Alphafuser* run.

mTERF9 is involved in regulating RNA metabolism and gene expression via direct interaction with ribosomal RNA (16S rRNA) and a number of different RNA binding and processing factors. It plays an important role in environmental response, RNA processing, splicing and the stability of chloroplast-encoded RNAs, including those necessary for the formation of photosynthetic complexes and chloroplast ribosome biogenesis (Méteignier *et al.*, 2021 ▸). As co-IP/MS will identify direct and indirect interactors of a target protein as well as a number of false positives, it is difficult to unambiguously identify direct protein complexes that may be of biological relevance. Based on experimental data, 39 highly enriched proteins from co-IP/MS experiments were selected for the generation of putative mTERF9 complexes in trimmed mode. These 39 proteins expanded to a total set of 780 trimeric complexes containing mTERF9. In the course of the calculation, 153 of these complexes were eliminated by failing priority scores below the 0.5 ipTM threshold and 627 were calculated (Supplementary Data S1).

**Figure 4:**
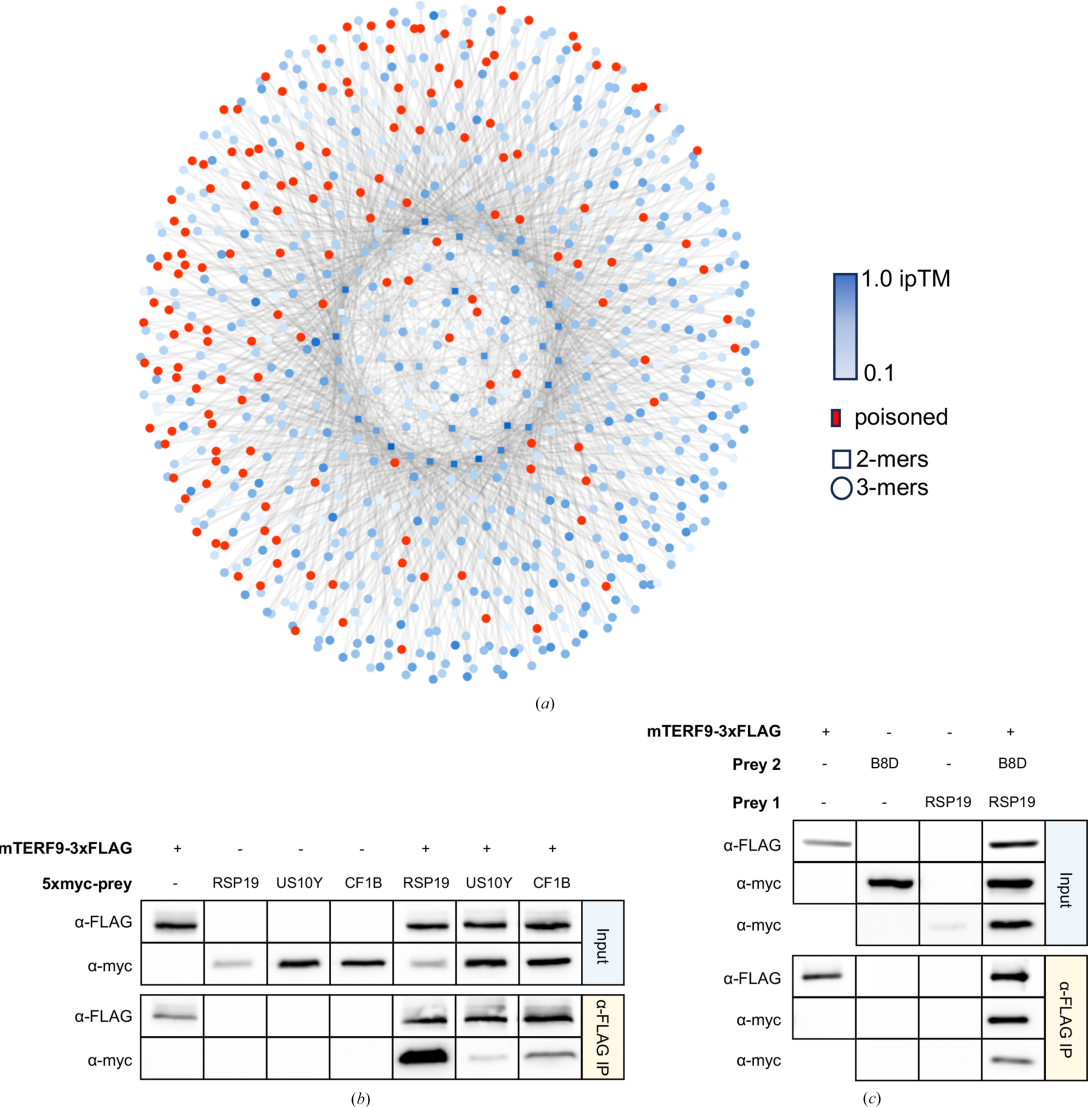
Network diagram and pull-down assays for the mTERF9 interactome. (*a*) Dimer, trimer and tetramer complexes queued for computation. Red denotes poisoned complexes based on an ipTM cutoff of 0.5. Dimers (squares), trimers (circles) and tetramers (triangles) are shown and are colored by ipTM, with darker blue denoting higher ipTM scores. (*b*) Pull-down experiment demonstrating interaction between high-scoring mTERF9 dimers. All proteins were expressed using *in vitro* transcription translation with an N-terminal 3×FLAG tag for mTERF9 and a 5×Myc tag for the different prey proteins. RSP, RSP19; US10, US10Y; CF1, ATHCF1β. (*c*) Pull-down experiment demonstrating interaction between an mTERF9 trimer. RSP19 was poorly expressed alone but was easily detectable when co-expressed and in the pull-down assay.

High-scoring mTERF9 complexes contained components of the chloroplast ribosome, including RSP19 and US10Y, proteins associated with the small chloroplast ribosomal 30S subunit, and RPL22, BL17C and EL30X, proteins associated with the large chloroplast ribosomal 50S subunit. Other high-scoring complexes contained proteins involved in stress response, such as peroxiredoxin Q (PRXQ) and ATRAB8D, and photosynthesis, including ATHCF1β, important for the assembly and stability of the chloroplast NAD(P)H dehydrogenase-like (NDH) complex, and CPFTSY, important for the targeting and integration of the light-harvesting chloro­phyll a/b-binding protein into the thylakoid membrane. As mentioned previously, complexes identified by co-IP mass spectrometry could be of a direct or indirect nature. Three of the dimers with high ipTM scores (>0.6), including the highest ipTM of 0.66 (mTERF9/RSP19), from the experimental dataset were tested and shown to interact (Fig. 4 ▸). ATRAB8D was cloned and the trimer consisting of ATRAB8D/mTERF9/RSP19 (ipTM 0.53) was tested, demonstrating a positive interaction (Fig. 4 ▸). Due to nonspecific interactions between the HA-tag and the anti-FLAG beads used in the pull-down assay, trimers with EL30X, CPFTSY and RPL22 (cloned with HA-tags) were excluded from the results.

## 4. Discussion

Structure-prediction pipelines based on AF demonstrate a high degree of overlap with experimental yeast two-hybrid data, suggesting that AF is a viable method for *in silico* yeast two-hybrid experiments (Lee *et al.*, 2024 ▸; Evans *et al.*, 2022 ▸; Bryant *et al.*, 2022 ▸; Kim *et al.*, 2024 ▸). Interestingly, when we compared AF predictions with *Arabidopsis* gynoecium TF interaction data, many of the complexes identified by yeast two-hybrid data exhibited low ipTM scores (<0.5). This includes the well studied and experimentally structurally validated dimeric and tetrameric MADS TF that were eliminated using the 0.5 ipTM cutoff at the binary-complex step (Puranik *et al.*, 2014 ▸; Hugouvieux *et al.*, 2018 ▸, 2024 ▸). This suggests that modifying the elimination parameters based on the interactome under consideration may be required to compute more physiologically relevant complexes. We experimentally validated the direct physical interaction of binary complexes with ipTM scores as low as 0.2, suggesting that a number of complexes would be eliminated due to the stringent ipTM cutoff of 0.5. Based on this, at least for the workflow presented here, it is easy to underestimate the number of higher-order complexes formed, as complex calculation depends on the ipTM cutoff criteria used. Based on both the TFIIIC complex example in the training set and the gynoecium data in the test set, ipTM scores in the range 0.2–0.4 may correspond to *bona fide* complexes. Improvement of confidence metrics, and/or the combination of different metrics, might improve the sensitivity of this method. Thus, while priority scores based on ipTM criteria provide a convenient way to limit the number of complexes computed, the results will favor well folded complexes with large surface-area contacts and will likely underestimate the number of true complexes due to their elimination during parsing based on ipTM score cutoff, particularly for highly disordered proteins with low pTM and pLDDT metrics. Based on the number of complexes to be computed and the available GPU, lower priority thresholds may be worth investigating with the caveat that the number of complexes to be generated and the output list of potential target complexes will be much higher. This is much more applicable to small interactomes and complexes of restricted size. While subject to limitations and with better performance using well folded interactomes, *Alphafuser* is a simple tool to implement in order to sort complexes and complement experimental interactome data for downstream experiments.

Expanding binary interactions to higher-order complexes in *Alphafuser* relies on the generation of a complete queue of complexes. One drawback to this approach is the large number of potential complexes that can be assembled from such an interaction list and the need to compute structures in a computationally parsimonious manner for practical implementation of the pipeline by the end user. The situation is somewhat improved by limiting the number of input proteins or the maximum number of proteins in a complex (in the cases examined here the complexes were all limited to three to four members), increasing the ipTM cutoff criteria as discussed above and/or implementing trimming protocols. It is also possible to filter binary interaction data for reciprocity, for example to only accept the *A*–*B* interaction if *A* is shown to interact with *B* and *B* is shown to interact with *A* to create a smaller starting set of interactions. For example, yeast two-hybrid experiments that consider as positive only binary interactions demonstrated in both bait and prey vectors may be used. It should be noted that the false-positive and false-negative rates for yeast two-hybrid experiments have been estimated to be ∼25% and ∼50%, respectively (Huang *et al.*, 2007 ▸). The high false-negative rate and reciprocity criteria will result in missing a number of true interactions as they will not be computed. The more stringent reciprocity criteria, however, will reduce the number of false positives and provide a high-confidence starting interactome.

Trimming protocols should be used with caution and in a target-specific manner, as shown here for the PP2A and gynoecium TF yeast two-hybrid dataset, where AF predicts a small and large percentage of residues with low confidence, respectively. Comparisons of the PP2A test case run in trimmed and untrimmed modes demonstrated a high overlap between the runs, suggesting that trimming protocols may be well adapted to some systems and allow faster computation. For proteins with extensive random coil, such as eukaryotic TFs, that may undergo co-folding upon complex formation, trimming will eliminate a high percentage of the primary sequence. In addition, the ipTM scores will be artificially inflated for trimmed structures and caution should be used when implementing trimming that removes over 50% of the polypeptide chain. In cases such as the *Arabidopsis* gynoecium TF interactome, where ipTM scores and pLDDT scores are low, it may be necessary to select more complexes for experimental testing, as well as applying more scrutiny to all available metrics such as pTM score and the PAE matrix as well as visual inspection of the pLDDT scores and structure-output thumbnails.

Through highly parallelized computing protocols, the generation and ranking of thousands of potential complexes may be performed in a reasonable time. Since *Alphafuser* generates a queue of all possible complexes to be computed as an initial step, dead-end elimination is an important aspect of the pipeline and results in the highly efficient removal of subcomplexes that fail a scoring step. The elimination steps propagate upwards in the queue throughout the run (*i.e.* will remove all higher-order complexes containing lower order complexes below the given threshold score). ipTM thresholds of 0.8–0.9, implemented for binary complexes, were generally suitable for training sets and offered a compromise between the number of structures generated and ranked versus the appearance of the ‘correct’ multimeric structure in the test cases used for parameterization for training cases. However, as discussed above, this parameter required adjustment for the yeast two-hybrid data and co-IP mass spectrometry data due to lower overall pTM scores for the monomeric structures and lower ipTM scores for the binary interactions, even for the experimentally validated yeast two-hybrid data, which, *a priori*, should represent true positives. As a rule of thumb, interactomes for proteins involved in core metabolic processes, enzymatic reaction pathways or plastid proteins, for example, are likely to be well folded and offer straightforward cases for fold prediction and complex prediction using the default *Alphafuser* pipeline parameters of a high threshold of 0.8–0.9 and optional trimming for faster computation. In contrast, transcriptional, signaling and stress-response pathways, for example, often contain flexible proteins with extensive disordered regions involved in dynamic interactions; as such, these interactomes are challenging targets for fold prediction. The *Alphafuser* priority threshold should be decreased (here to 0.5 for TF complexes) and trimming parameters should be used with caution and generally avoided if the target protein or a large percentage of its interactome have greater than 40–50% predicted disorder.

*Alphafuser* ranks complexes based on ipTM score and includes a script for generating a thumbnail output in *PyMOL* (Schrödinger) for easy visualization and manual inspection, providing a quick and intuitive method to parse *Alphafuser* output. In addition, as with any *AlphaFold* prediction, each structure generated includes the PAE. While ipTM specifically measures the confidence of the protein–protein interface, the pLDDT and pTM provide important complementary information about local structural accuracy and global topology, which should be considered by the user when evaluating the final *Alphafuser* output. Other metrics should be incorporated by the end user prior to downstream studies of a putative complex, such as the amount of buried surface area upon complex formation, pDockQ or calculations of free energy for individual components and complexes (Krissinel & Henrick, 2007 ▸; Ribeiro *et al.*, 2019 ▸; Burke *et al.*, 2023 ▸). Considering all of these metrics reduces false positives and allows a more nuanced evaluation of whether a predicted protein complex is likely to form.

In default mode, the stoichiometry of complexes is assumed to be 1:1 for all components, thus neglecting homo-oligomers. However, if the stoichiometry of one or more members of a complex is known, for example an obligate homodimer is present for a protein, this can be easily incorporated by repeating the sequence of interest in the database generated during the *Alphafuser* run (via SQL) and will increase the utility of the pipeline in identifying novel and putatively physiologically relevant protein complexes. It should be noted that protein complexes that form in a cellular context will do so under cellular conditions (*i.e.* specific temperatures, pH, molecular crowding *etc.*; Brink *et al.*, 2025 ▸). AF does not mimic or simulate these conditions and consequently the complexes identified in our pipeline, while ranked by probability scores, may or may not represent *in vivo* assemblies. However, by combining experimental interaction data with prediction, we provide a starting point for further investigation of the most likely assemblies.

## 5. Conclusions

Predicting protein folding and the probability of forming multimeric protein complexes are major advances in structural biology, providing not only testable structural insights but also clues into the details of the interactome underlying the proper function of cellular processes. *AlphaFold* has been trained on static structures at cryogenic temperatures, which are potentially nonphysiological, and significant work has been performed recently to address the question of dynamics (Zhu *et al.*, 2023 ▸; Janson *et al.*, 2025 ▸; Cui *et al.*, 2025 ▸). These advances offer exciting potential improvements to the *Alphafuser* approach, because the method is largely agnostic to the type of fold prediction that is used (as long as some form of reliable confidence metric is produced). By the same token, future improvements to scoring (usually included in the fold prediction itself, but potentially decoupled as well) could offer improvements to accuracy of *Alphafuser*. As protein fold-prediction methods and high-quality experimental interactome datasets continue to expand, tools such as *Alphafuser* will become increasingly powerful for systematically exploring protein–protein interaction networks and prioritizing potentially biologically relevant multiprotein assemblies for experimental validation.

## Supplementary Material

Supplementary Table S1. DOI: 10.1107/S2059798326003013/nw5134sup1.pdf

Supplementary Data S1. ipTM and status for test and experimental systems. DOI: 10.1107/S2059798326003013/nw5134sup2.xlsx

## Figures and Tables

**Table 1:** Summary of training complexes used

Complex	BioGRID bait	Selection criteria	Maximum complex size	Total No. of complexes
PP2A	Protein phosphatase II, regulatory subunit A	Affinity capture	4	63
TFIIIC complex	General transcription factor IIIC, polypeptide 6, α, 35 kDa	Affinity capture	4	468
TRAPP I complex	TRAPPC3 trafficking protein particle complex 3	Affinity capture	4	1156
Human γ-secretase complex	Aph1a γ-secretase subunit	Affinity capture	4	469

**Table 2:** Summary of ipTM thresholds and *Alphafuser* complexes computed for test cases

Priority threshold	Completed complexes	Poisoned complexes	Solution based on PDB
PP2A			
0.5	52	10	Present
0.6	52	10	Present
0.7	52	10	Present
0.8	47	16	Present
0.9	37	26	Present
TFIIIC			
0.5	178	286	Present
0.6	39	430	Eliminated
0.7	39	430	Eliminated
0.8	39	430	Eliminated
0.9	39	430	Eliminated
TRAPP			
0.5	912	220	Present
0.6	804	341	Present
0.7	729	419	Present
0.8	646	510	Present
0.9	135	1024	Eliminated
γ-Secretase			
0.5	249	220	Present
0.6	132	337	Present
0.7	132	337	Present
0.8	128	341	Present
0.9	50	419	Eliminated
