# Supplementary material for: Alphafuser: a parsimonious approach to predicting higher-order protein complexes
Source: Acta Crystallogr D Struct Biol. 2026 Apr 23;82(Pt 5):421–33. doi: 10.1107/S2059798326003013 (PMC13133988; doi:10.1107/S2059798326003013)
Supplement: Supplementary file 1 [file d-82-00421-sup1.pdf]

STRUCTURAL
BIOLOGY

Volume 82 (2026)

Supporting information for article:

Alphafuser: a parsimonious approach to predicting higher-order protein complexes

Audrey Guillotin, Stephanie Hutin, Lorelei Masselot-Joubert, Kamel Hammani, Chloe Zubieta and Max Nanao

Table S1 Primer list for pTnT constructs

Gynoecium Interactors	Accession number	Tag	Primers
**AS2**	AT1G65620	C-3xFLAG	ACGGTAGCGGATCTGGGAGCATGGCATCTTCTTCAACAAACTCACC
			TCGTCGTCCTTATAATCggaAGACGGATCAACAGTACGGCG
**BEE1**	AT1G18400	C-3xFLAG	ACGGTAGCGGATCTGGGAGCATGGCAAATTTCGAGAATCTTTCTTCTG
			TCGTCGTCCTTATAATCggaAAGGGACCATGTTGATAAATGGAAGAC
		C-3xHA	CGCGTGGTACCTCTAGAGTCATGGCAAATTTCGAGAATCTTTCTTCTGA
			tacagctcgtccatgccGTCAAGGGACCATGTTGATAAATGGAAGAC
		N-StrepTagII	TCGAAAAAGGAAGCGGCTCAATGGCAAATTTCGAGAATCTTTCTTCTG
			CGCCCGGGTCGACTCTAGAGTCAAAGGGACCATGTTGATAAATGGAAG
**BP**	AT4G08150	C-3xHA	CGCGTGGTACCTCTAGAGTCATGGAAGAATACCAGCATGACAACAGC
			tacagctcgtccatgccGTCTGGACCGAGACGATAAGGTCCATC
		N-V5	ATTCTACGGGAAGCGGCTCAATGGAAGAATACCAGCATGACAACAG
			CCCGGGTCGACTCTAGAGGTTTATGGACCGAGACGATAAGGTCC
**CUC1 (non-amplified from cDNA)**	AT3G15170	C-3xFLAG	ACGGTAGCGGATCTGGGAGCATGGATGTTGATGTGTTTAACGGTTGGG
			TCGTCGTCCTTATAATCggaGAGAGTAAACGGCCACACACTCAC
		C-3xHA	CGCGTGGTACCTCTAGAGTCATGGATGTTGATGTGTTTAACGGTTGG
			tacagctcgtccatgccGTCGAGAGTAAACGGCCACACACTC
**FIL**	AT2G45190	C-3xFLAG	ACGGTAGCGGATCTGGGAGCATGTCTATGTCGTCTATGTCCTCCC
			TCGTCGTCCTTATAATCggaATAAGGAGTCACACCAACGTTAGC
**HEC1**	AT5G67060	N-5xMyc	AAGAGGACTTGAATGAAATGGATTCTGACATAATGAACATGATGATGC
			TTAGAGGCCCCAAGGGGTTATCTAAGAATCTGTGCATTGCCCACC

**HEC2**	AT3G50330	C-3xHA	CGCGTGGTACCTCTAGAGTCATGGATAACTCCGACATTCTAATGAACATGATG
			tacagctcgtccatgccGTCTCTAAGAATCTGTGCATTTCCAAGCATCTG
**IND**	AT4G00120	N-V5	ATTCTACGGGAAGCGGCTCAATGGAAAATGGTATGTATAAAAAGAAAGGAGTGTG
			CCCGGGTCGACTCTAGAGGTTCAGGGTTGGGAGTTGTGGTAATAAC
**KAN2**	AT1G32240	C-3xFLAG	ACGGTAGCGGATCTGGGAGCATGGAGCTGTTTCCTGCTCAGC
			TCGTCGTCCTTATAATCggaGTGAGATCGACCCAGAGTAAACTCAAG
**KNAT6**	AT1G23380	N-StrepTagII	TCGAAAAAGGAAGCGGCTCAATGGATGGAATGTACAATTTCCATTCGG
			CGCCCGGGTCGACTCTAGAGTCATTCCTCGGTAAAGAATGATCCACTAGAATC
**LUG (non-amplified from cDNA)**	AT4G32551	N-StrepTagII	TCGAAAAAGGAAGCGGCTCAATGTCTCAGACCAACTGGG
			CGCCCGGGTCGACTCTAGAGTCACTTCCACAGTTTCACTAGCTTATCATGACT
**NGA3**	AT1G01030	N-StrepTagII	TCGAAAAAGGAAGCGGCTCAATGGATCTATCCCTGGCTCC
			CGCCCGGGTCGACTCTAGAGTCATGGATTGAAATTGAGAGAAAGTGAAGACTTC
**PNF**	AT2G27990	N-5xMyc	AAGAGGACTTGAATGAAATGATGGATATGATAAAACCAGATTTTCAACAAATCCGAAG
			TTAGAGGCCCCAAGGGGTTAACCCACAAAGTCATGAAACATTTGGC
**RPL**	AT5G02030	C-3xHA	CGCGTGGTACCTCTAGAGTCATGGCTGATGCATACGAGCC
			tacagctcgtccatgccGTCACCTACAAAATCATGTAGAAACTGATGATTACTACC
		N-5xMyc	AAGAGGACTTGAATGAAATGATGGCTGATGCATACGAGCC
			TCTAGAGGTACCACCCGCTCTCAACCTACAAAATCATGTAGAAACTGATGATTACTAC
**SPT**	AT4G36930	N-StrepTagII	TCGAAAAAGGAAGCGGCTCAATGATATCACAGAGAGAAGAAAGAGAAGAGAAGAAGC
			CGCCCGGGTCGACTCTAGAGTCAAGTAATTCGATCTTTTAGGTCAGGTTGTCC
**STM**	AT1G62360	C-3xFLAG	ACGGTAGCGGATCTGGGAGCATGGAGAGTGGTTCCAACAGC
			TCGTCGTCCTTATAATCggaAAGCATGGTGGAGGAGATGTG
**WUS**	AT2G17950	N-V5	ATTCTACGGGAAGCGGCTCAATGGAGCCGCCACAGC

			CCCGGGTCGACTCTAGAGGTCTAGTTCAGACGTAGCTCAAGAGAAGC
**YAB3**	AT4G00180	N-StrepTagII	TCGAAAAAGGAAGCGGCTCAATGTCGAGCATGTCCATGTCG
			CGCCCGGGTCGACTCTAGAGCTAGTTATGGGCCACCCCAAC
**mTERF9 Interactors**	**Accession number**	**Tag**	**Primers**
**ATHCF1BETA**	ATCG00480	N-5xMyc	AAGAGGACTTGAATGAAATGATGAGAACAAATCCTACTACTTCAAATCCAGAG
			TCTAGAGGTACCACCCGCTCTCATTTCTTCAATTTACTCTCCATTTCTAAGTTCG
**ATRAB8D**	AT4G20360	N-5xMyc	AAGAGGACTTGAATGAAATGATGGCGATTTCGGCTCCAG
			TCTAGAGGTACCACCCGCTCTCATTCGAGGATCGTCCCAATAACTC
**BL17C**	AT3G54210	C-3xHA	CGCGTGGTACCTCTAGAGTCATGGCGATTCCAATGTCCATGG
			TACAGCTCGTCCATGCCGTCGACAAGCTCAATGTAAGCCATTGGAG
		N-5xMyc	AAGAGGACTTGAATGAAATGATGGCGATTCCAATGTCCATGG
			TCTAGAGGTACCACCCGCTCTTAGACAAGCTCAATGTAAGCCATTGG
**CPFTSY**	AT2G45770	C-3xHA	CGCGTGGTACCTCTAGAGTCATGGCAACTTCTTCTGCTCACC
			TACAGCTCGTCCATGCCGTCAGAGAATATAGCATTCACAAAAGCCTCC
**EL30X**	AT3G18740	C-3xHA	CGCGTGGTACCTCTAGAGTCATGGTTGCGGAGAAGAAGGC
			TACAGCTCGTCCATGCCGTCCTGATCTCCAGGAAGTGTCTTGATGATG
**mTERF9**	AT5G55580	C-3xFLAG	TCTAGAGGTACCACCCGCTCTCATTTCTTCAATTTACTCTCCATTTCTAAGTTCG
			TCGTCGTCCTTATAATCGGATCCTCTCTTGTCATACTTGTTTGCAAAC
**P2Y (no expression by TnT)**	AT2G27710	N-5xMyc	AAGAGGACTTGAATGAAATGATGAAGGTTGTTGCCGCATAC
			TCTAGAGGTACCACCCGCTCTTACTCGAATAGACTGAAACCCATGTCATC
**PXRQ**	AT3G26060	N-5xMyc	AAGAGGACTTGAATGAAATGATGGCTGCTTCATCTTCCTCTTTC
			TCTAGAGGTACCACCCGCTCTCAAGCAGCTTTGAGAAACTTCAAGG
**RPL22**	ATCG00810	C-3xHA	CGCGTGGTACCTCTAGAGTCATGATAAAGAAGAGAAAAAAGAAATCATATACGGAAGTATATGC

			TACAGCTCGTCCATGCCGTCTTTTTTGTCCCATAGGCCTCCAC TAC
**RSP19**	ATCG00820	N-4xMyc	AAGAGGACTTGAATGAAATGGTGACACGTTCACTAAAAAAA AACCCTTTTG
			TCTAGAGGTACCACCCGCTCTTAACGACGAGATCTATTATCA TTTTTGGCATGT
**US10Y**	AT3G47370	N-5xMyc	AAGAGGACTTGAATGAAATGATGGCGTATGAACCGATGAAG C
			TCTAGAGGTACCACCCGCTCCTAAGAGTCAGCAATAGTGACC TCGAC
